# Supplementary material for: Mechanisms governing the pioneering and redistribution capabilities of the non-classical pioneer PU.1
Source: Nat Commun. 2020 Jan 21;11:402. doi: 10.1038/s41467-019-13960-2 (PMC6972792; doi:10.1038/s41467-019-13960-2)
Supplement: Supplementary file 7 — Source data [file 41467_2019_13960_MOESM7_ESM.zip › Source_Data/FigureS3/FigureS3H_MotifScanOutput/homerResults.html]

/misc/data/analysis/project\_PU1/predictionTALL1/motifs/ATAC\_PU1mutvsPU1.peaks.DESEQ/final - Homer de novo Motif Results


# Homer *de novo* Motif Results (/misc/data/analysis/project\_PU1/predictionTALL1/motifs/ATAC\_PU1mutvsPU1.peaks.DESEQ/final)

Known Motif Enrichment Results  
Gene Ontology Enrichment Results  
If Homer is having trouble matching a motif to a known motif, try copy/pasting the matrix file into
STAMP  
More information on motif finding results: HOMER
| Description of Results
| Tips
  
Total target sequences = 3866  
Total background sequences = 44808  
\* - possible false positive  

|  |  |  |  |  |  |  |  |  |
| --- | --- | --- | --- | --- | --- | --- | --- | --- |
| Rank | Motif | P-value | log P-pvalue | % of Targets | % of Background | STD(Bg STD) | Best Match/Details | Motif File |
| 1 | C A G T A C G T G A C T C A G T A C T G G C A T A C T G C T A G A G C T G A C T C G A T A G T C | 1e-659 | -1.518e+03 | 51.32% | 12.38% | 213.7bp (142.0bp) | RUNX1(Runt)/Jurkat-RUNX1-ChIP-Seq(GSE29180)/Homer(0.988) More Information \| Similar Motifs Found | motif file (matrix) |
| 2 | A T C G T C G A G T A C C G T A A C T G A C T G C G T A C G T A C T A G A G C T T A C G C T A G | 1e-286 | -6.589e+02 | 26.36% | 6.49% | 241.8bp (139.8bp) | ERG(ETS)/VCaP-ERG-ChIP-Seq(GSE14097)/Homer(0.954) More Information \| Similar Motifs Found | motif file (matrix) |
| 3 | A C T G G C T A A C T G A T C G A C G T C G A T C G A T | 1e-190 | -4.386e+02 | 46.64% | 23.80% | 280.6bp (140.0bp) | IRF4(IRF)/GM12878-IRF4-ChIP-Seq(GSE32465)/Homer(0.769) More Information \| Similar Motifs Found | motif file (matrix) |
| 4 | G T C A A C T G A C T G C G T A A C G T A C T G A G C T A C T G A T C G A C T G | 1e-136 | -3.150e+02 | 7.17% | 0.76% | 193.1bp (127.3bp) | ETS:RUNX(ETS,Runt)/Jurkat-RUNX1-ChIP-Seq(GSE17954)/Homer(0.940) More Information \| Similar Motifs Found | motif file (matrix) |
| 5 | A G T C A C G T A C G T A G T C A G T C A C T G A C T G | 1e-132 | -3.043e+02 | 47.70% | 28.18% | 284.1bp (138.4bp) | ELF1(ETS)/Jurkat-ELF1-ChIP-Seq(SRA014231)/Homer(0.953) More Information \| Similar Motifs Found | motif file (matrix) |
| 6 | T G C A G C T A T C A G A G T C C G T A A T C G A T G C C G A T A C T G A G T C C A G T | 1e-130 | -3.006e+02 | 24.29% | 9.94% | 267.0bp (151.3bp) | E2A(bHLH)/proBcell-E2A-ChIP-Seq(GSE21978)/Homer(0.969) More Information \| Similar Motifs Found | motif file (matrix) |
| 7 | T A G C G A C T A C G T G C A T T C A G C T G A C G A T T A C G C A G T C T A G C T A G | 1e-120 | -2.770e+02 | 16.09% | 5.17% | 237.3bp (148.0bp) | Tcf4(HMG)/Hct116-Tcf4-ChIP-Seq(SRA012054)/Homer(0.830) More Information \| Similar Motifs Found | motif file (matrix) |
| 8 | A G T C A C G T A C T G A G T C T A C G A C T G A C G T A C G T | 1e-110 | -2.544e+02 | 45.03% | 27.38% | 309.4bp (142.5bp) | RUNX2/MA0511.2/Jaspar(0.919) More Information \| Similar Motifs Found | motif file (matrix) |
| 9 | C G T A C G T A T G A C A G T C T A C G G T C A G T C A C G T A A T C G G A C T | 1e-83 | -1.915e+02 | 20.54% | 9.58% | 299.0bp (140.1bp) | MF0009.1\_TRP(MYB)\_class/Jaspar(0.872) More Information \| Similar Motifs Found | motif file (matrix) |
| 10 | A G C T G A T C C T G A A T G C A C G T A C G T A C G T A G T C C G T A A G T C C G A T A C G T A G C T A G T C | 1e-77 | -1.785e+02 | 4.50% | 0.57% | 189.4bp (131.1bp) | IRF1(IRF)/PBMC-IRF1-ChIP-Seq(GSE43036)/Homer(0.967) More Information \| Similar Motifs Found | motif file (matrix) |
| 11 | G C A T C A T G G C A T C A T G C T A G A G C T A G C T A C G T T G A C G A T C G C A T A T C G | 1e-73 | -1.700e+02 | 9.60% | 2.96% | 212.6bp (137.2bp) | EWS:FLI1-fusion(ETS)/SK\_N\_MC-EWS:FLI1-ChIP-Seq(SRA014231)/Homer(0.754) More Information \| Similar Motifs Found | motif file (matrix) |
| 12 | T C A G C G T A G A T C C A G T A C T G A G T C A C T G A C T G A G T C G A C T A T C G G C A T A G C T | 1e-73 | -1.692e+02 | 41.93% | 27.66% | 309.2bp (145.8bp) | RUNX1(Runt)/Jurkat-RUNX1-ChIP-Seq(GSE29180)/Homer(0.741) More Information \| Similar Motifs Found | motif file (matrix) |
| 13 | A G C T A G C T T C G A T A G C G T A C C G T A A T G C C G T A T C A G T G C A T G A C T A G C | 1e-68 | -1.588e+02 | 18.96% | 9.23% | 279.5bp (142.8bp) | RUNX2(Runt)/PCa-RUNX2-ChIP-Seq(GSE33889)/Homer(0.829) More Information \| Similar Motifs Found | motif file (matrix) |
| 14 | C A G T T C A G A T G C A C G T A G C T C A G T A T G C C G A T A T C G C G T A | 1e-62 | -1.443e+02 | 25.53% | 14.67% | 335.2bp (139.9bp) | IRF4(IRF)/GM12878-IRF4-ChIP-Seq(GSE32465)/Homer(0.814) More Information \| Similar Motifs Found | motif file (matrix) |
| 15 | C T A G T A G C C G T A A T C G C G A T A C T G C G A T C A T G C A T G A C G T A C G T A C G T | 1e-57 | -1.314e+02 | 20.02% | 10.77% | 259.9bp (146.2bp) | RUNX1(Runt)/Jurkat-RUNX1-ChIP-Seq(GSE29180)/Homer(0.714) More Information \| Similar Motifs Found | motif file (matrix) |
| 16 | G C A T C T A G G T A C A G T C C G T A G A T C G T A C C G A T A T C G G A C T | 1e-56 | -1.292e+02 | 22.84% | 13.03% | 309.2bp (143.2bp) | FIGLA/MA0820.1/Jaspar(0.875) More Information \| Similar Motifs Found | motif file (matrix) |
| 17 | G T A C A C T G A C T G G T C A C G T A C G T A A C G T A T C G | 1e-49 | -1.148e+02 | 9.98% | 4.07% | 297.0bp (133.5bp) | Fli1(ETS)/CD8-FLI-ChIP-Seq(GSE20898)/Homer(0.763) More Information \| Similar Motifs Found | motif file (matrix) |
| 18 | T G A C G C T A T C A G A T G C C G A T A T C G T A G C C G T A A T C G T A G C G C A T A C T G A G C T A C G T | 1e-47 | -1.098e+02 | 37.35% | 26.16% | 305.5bp (150.8bp) | PB0003.1\_Ascl2\_1/Jaspar(0.764) More Information \| Similar Motifs Found | motif file (matrix) |
| 19 | A T G C A G T C A C G T A G C T C G A T T A C G G T C A C G A T | 1e-43 | -1.006e+02 | 25.30% | 16.09% | 270.8bp (138.0bp) | Tcf3(HMG)/mES-Tcf3-ChIP-Seq(GSE11724)/Homer(0.926) More Information \| Similar Motifs Found | motif file (matrix) |
| 20 | G A T C G A T C A G T C G A T C G A T C C G A T A T G C G A T C G A T C G A T C G A T C G A T C A G T C A G T C | 1e-39 | -9.069e+01 | 14.33% | 7.72% | 346.4bp (128.7bp) | Zfp281(Zf)/ES-Zfp281-ChIP-Seq(GSE81042)/Homer(0.870) More Information \| Similar Motifs Found | motif file (matrix) |
| 21 | T G C A C G T A T A C G A T G C T A C G C A G T C A T G T A G C G A C T A C G T G A C T | 1e-39 | -9.037e+01 | 18.03% | 10.58% | 283.9bp (140.2bp) | Ahr::Arnt/MA0006.1/Jaspar(0.609) More Information \| Similar Motifs Found | motif file (matrix) |
| 22 | C T A G T C A G A G T C C G A T A T C G A C G T A T G C C G T A A C T G T A G C | 1e-38 | -8.892e+01 | 33.47% | 23.73% | 325.4bp (146.7bp) | Meis1(Homeobox)/MastCells-Meis1-ChIP-Seq(GSE48085)/Homer(0.817) More Information \| Similar Motifs Found | motif file (matrix) |
| 23 | C A T G C A T G G A T C G A C T A G C T C G A T A T G C G C T A T A G C C G T A A T C G G T C A | 1e-37 | -8.566e+01 | 19.81% | 12.19% | 316.5bp (143.9bp) | Rbpj1(?)/Panc1-Rbpj1-ChIP-Seq(GSE47459)/Homer(0.631) More Information \| Similar Motifs Found | motif file (matrix) |
| 24 | C G T A T C A G G T A C G T C A T G A C C T G A T A C G G T A C C G A T A C T G A G T C A G C T A G C T A G C T | 1e-37 | -8.521e+01 | 13.11% | 6.98% | 336.1bp (144.2bp) | Tcf12(bHLH)/GM12878-Tcf12-ChIP-Seq(GSE32465)/Homer(0.757) More Information \| Similar Motifs Found | motif file (matrix) |
| 25 | C G T A C T A G G A T C C G A T T A C G C G T A C T A G A G T C G C A T T A C G G C A T | 1e-34 | -7.977e+01 | 31.45% | 22.42% | 326.0bp (147.7bp) | Ap4(bHLH)/AML-Tfap4-ChIP-Seq(GSE45738)/Homer(0.635) More Information \| Similar Motifs Found | motif file (matrix) |
| 26 | T C G A C G A T T A C G C G T A A T C G A C G T T G A C T C G A A C G T G C T A A G T C | 1e-31 | -7.231e+01 | 7.58% | 3.39% | 231.0bp (136.8bp) | BATF(bZIP)/Th17-BATF-ChIP-Seq(GSE39756)/Homer(0.968) More Information \| Similar Motifs Found | motif file (matrix) |
| 27 | G C A T A C G T A T C G A T G C G C A T C A G T C G A T C T A G C G T A C G T A T C A G G C A T | 1e-30 | -6.958e+01 | 5.79% | 2.30% | 276.7bp (144.9bp) | Tcf3(HMG)/mES-Tcf3-ChIP-Seq(GSE11724)/Homer(0.824) More Information \| Similar Motifs Found | motif file (matrix) |
| 28 | A G C T C G A T G T C A G C T A T C A G A G T C G C A T A T C G A G T C | 1e-29 | -6.804e+01 | 17.64% | 11.15% | 293.7bp (146.5bp) | SCL(bHLH)/HPC7-Scl-ChIP-Seq(GSE13511)/Homer(0.686) More Information \| Similar Motifs Found | motif file (matrix) |
| 29 | A C T G A C T G A T G C G C A T A T C G A G T C A G C T C T G A G T C A | 1e-27 | -6.372e+01 | 32.36% | 24.17% | 319.5bp (143.9bp) | POL010.1\_DCE\_S\_III/Jaspar(0.761) More Information \| Similar Motifs Found | motif file (matrix) |
| 30 | A C G T A C T G C G T A T A G C A G T C A G C T C G T A A T G C C G A T A C G T A G C T | 1e-27 | -6.221e+01 | 1.86% | 0.31% | 180.9bp (125.5bp) | RORA(var.2)/MA0072.1/Jaspar(0.929) More Information \| Similar Motifs Found | motif file (matrix) |
| 31 | C T A G A G T C A C G T A C T G C G A T C T A G A T G C C G T A A T G C G C T A | 1e-25 | -5.918e+01 | 38.02% | 29.71% | 303.5bp (145.9bp) | POL009.1\_DCE\_S\_II/Jaspar(0.811) More Information \| Similar Motifs Found | motif file (matrix) |
| 32 | A C T G C G T A C G A T A C G T C T A G A G C T A C G T C G T A A C G T A G T C A G C T | 1e-25 | -5.842e+01 | 0.91% | 0.04% | 173.7bp (107.7bp) | GATA(Zf),IR4/iTreg-Gata3-ChIP-Seq(GSE20898)/Homer(0.807) More Information \| Similar Motifs Found | motif file (matrix) |
| 33 | A T G C A G T C A C G T A T C G G C A T A C T G A T C G A G T C C G A T A C T G A G T C G C A T A C T G T G A C | 1e-24 | -5.749e+01 | 21.47% | 14.88% | 298.3bp (145.4bp) | MafK(bZIP)/C2C12-MafK-ChIP-Seq(GSE36030)/Homer(0.570) More Information \| Similar Motifs Found | motif file (matrix) |
| 34 | A G T C A C T G A C G T C T A G A C T G A C T G G T C A | 1e-24 | -5.606e+01 | 41.31% | 33.04% | 339.1bp (139.9bp) | Rbpj1(?)/Panc1-Rbpj1-ChIP-Seq(GSE47459)/Homer(0.826) More Information \| Similar Motifs Found | motif file (matrix) |
| 35 | C T G A A G T C T G A C A T C G A C T G C G T A A C G T | 1e-24 | -5.560e+01 | 11.25% | 6.56% | 277.5bp (134.3bp) | SPDEF/MA0686.1/Jaspar(0.692) More Information \| Similar Motifs Found | motif file (matrix) |
| 36 | A C T G C T G A A C G T A G T C A C G T C G T A A T C G A G T C A G T C C G T A A G T C A G T C A G T C | 1e-23 | -5.441e+01 | 0.72% | 0.02% | 233.9bp (105.0bp) | PB0167.1\_Sox13\_2/Jaspar(0.606) More Information \| Similar Motifs Found | motif file (matrix) |
| 37 | A C G T A G T C A C G T C G T A A T C G A G T C G T A C G T C A G C A T A G T C A G T C G T C A A C T G A G T C | 1e-23 | -5.363e+01 | 0.65% | 0.01% | 149.7bp (41.2bp) | SMAD3/MA0795.1/Jaspar(0.575) More Information \| Similar Motifs Found | motif file (matrix) |
| 38 | T C A G C A T G C A T G C A T G A C T G A G C T C A G T C A T G C A T G A C T G A G T C C G A T | 1e-23 | -5.355e+01 | 6.98% | 3.45% | 403.1bp (139.8bp) | POL003.1\_GC-box/Jaspar(0.712) More Information \| Similar Motifs Found | motif file (matrix) |
| 39 | C T A G C G T A A C G T C G T A T G C A T A G C C T G A A C G T A G T C C G A T | 1e-22 | -5.284e+01 | 2.46% | 0.65% | 228.7bp (121.7bp) | PB0125.1\_Gata3\_2/Jaspar(0.871) More Information \| Similar Motifs Found | motif file (matrix) |
| 40 | C T A G G T A C C T G A A T G C C G T A T C A G G C A T G T C A C T A G A C G T G C T A T A G C | 1e-22 | -5.253e+01 | 1.37% | 0.19% | 171.5bp (162.4bp) | PB0208.1\_Zscan4\_2/Jaspar(0.735) More Information \| Similar Motifs Found | motif file (matrix) |
| 41 | G T A C C G T A A G T C A T G C C A G T C T A G T A G C C T A G | 1e-22 | -5.250e+01 | 10.48% | 6.08% | 290.3bp (162.2bp) | Slug(Zf)/Mesoderm-Snai2-ChIP-Seq(GSE61475)/Homer(0.789) More Information \| Similar Motifs Found | motif file (matrix) |
| 42 | C A T G T C A G T C A G C T A G A C T G A T G C A G T C A G T C A G T C | 1e-22 | -5.233e+01 | 13.14% | 8.18% | 367.4bp (133.5bp) | ZNF692(Zf)/HEK293-ZNF692.GFP-ChIP-Seq(GSE58341)/Homer(0.811) More Information \| Similar Motifs Found | motif file (matrix) |
| 43 | A C T G A T C G T C A G C T A G T C A G C T A G A C T G | 1e-22 | -5.220e+01 | 20.85% | 14.64% | 371.2bp (130.7bp) | ZNF740/MA0753.1/Jaspar(0.904) More Information \| Similar Motifs Found | motif file (matrix) |
| 44 | G T C A A T G C C T G A C T G A G T C A C G T A A C T G C T G A T G A C A C G T A G T C A G C T | 1e-22 | -5.116e+01 | 0.83% | 0.04% | 174.1bp (140.1bp) | Sox9(HMG)/Limb-SOX9-ChIP-Seq(GSE73225)/Homer(0.671) More Information \| Similar Motifs Found | motif file (matrix) |
| 45 | A C G T A C T G A C G T A G T C A C G T A C T G A G T C C G T A G T A C C G T A A C T G C G T A C T A G | 1e-21 | -5.035e+01 | 0.65% | 0.02% | 149.9bp (52.8bp) | Fox:Ebox(Forkhead,bHLH)/Panc1-Foxa2-ChIP-Seq(GSE47459)/Homer(0.678) More Information \| Similar Motifs Found | motif file (matrix) |
| 46 | G T A C A C G T A T G C G C A T A T C G C G T A T C A G G T A C A C T G | 1e-21 | -4.992e+01 | 40.43% | 32.67% | 321.0bp (146.5bp) | POL002.1\_INR/Jaspar(0.615) More Information \| Similar Motifs Found | motif file (matrix) |
| 47 | T C A G A G C T C G A T C G A T T G A C T G A C C G A T C G A T A G C T | 1e-20 | -4.823e+01 | 5.69% | 2.70% | 318.0bp (133.2bp) | PB0033.1\_Irf3\_1/Jaspar(0.836) More Information \| Similar Motifs Found | motif file (matrix) |
| 48 | A G C T C G A T G T A C C G A T A G T C C G T A A T C G C T G A | 1e-20 | -4.714e+01 | 26.36% | 19.83% | 332.6bp (145.8bp) | Rbpj1(?)/Panc1-Rbpj1-ChIP-Seq(GSE47459)/Homer(0.800) More Information \| Similar Motifs Found | motif file (matrix) |
| 49 | A C G T T A G C T C G A A C T G A C G T A G C T C G T A | 1e-20 | -4.707e+01 | 29.05% | 22.27% | 289.6bp (140.2bp) | FOS::JUN/MA0099.2/Jaspar(0.762) More Information \| Similar Motifs Found | motif file (matrix) |
| 50 | A C G T A C T G A C G T C T A G A T C G C G A T A C T G A G T C A G C T | 1e-19 | -4.596e+01 | 33.68% | 26.62% | 300.4bp (147.4bp) | RUNX2(Runt)/PCa-RUNX2-ChIP-Seq(GSE33889)/Homer(0.692) More Information \| Similar Motifs Found | motif file (matrix) |
| 51 | C A T G A C G T A C G T A T G C A G T C A G T C G A C T A C G T A C T G G C T A C G A T C A T G G A C T G C T A | 1e-19 | -4.583e+01 | 0.80% | 0.05% | 210.8bp (86.7bp) | PB0083.1\_Tcf7\_1/Jaspar(0.715) More Information \| Similar Motifs Found | motif file (matrix) |
| 52 | A G C T A C G T A G T C C G A T C T G A A C T G C G T A A C G T A G T C A C G T C G T A A C T G | 1e-19 | -4.571e+01 | 0.59% | 0.01% | 129.4bp (48.8bp) | PB0090.1\_Zbtb12\_1/Jaspar(0.701) More Information \| Similar Motifs Found | motif file (matrix) |
| 53 | C T A G C G T A C T A G A G T C G C A T A T C G A G C T C G A T | 1e-19 | -4.469e+01 | 20.36% | 14.66% | 294.3bp (145.4bp) | MyoG(bHLH)/C2C12-MyoG-ChIP-Seq(GSE36024)/Homer(0.759) More Information \| Similar Motifs Found | motif file (matrix) |
| 54 | A C T G A C T G A C T G T G A C A G C T A C G T A C T G G A C T A G T C A G C T A C T G A G T C | 1e-19 | -4.435e+01 | 0.80% | 0.06% | 280.6bp (136.5bp) | Smad3(MAD)/NPC-Smad3-ChIP-Seq(GSE36673)/Homer(0.623) More Information \| Similar Motifs Found | motif file (matrix) |
| 55 | C G T A C T A G A G T C G A T C T C G A G T C A C T G A G T A C G A C T A T C G A G C T C T A G C A T G C A G T | 1e-19 | -4.414e+01 | 0.67% | 0.03% | 119.5bp (115.1bp) | RUNX-AML(Runt)/CD4+-PolII-ChIP-Seq(Barski\_et\_al.)/Homer(0.617) More Information \| Similar Motifs Found | motif file (matrix) |
| 56 | A T C G C G A T A C T G A C T G A G C T A G C T A C G T A G C T C A T G A C T G A G C T A G C T C A G T A G T C | 1e-18 | -4.290e+01 | 1.50% | 0.30% | 372.0bp (123.7bp) | IRF8/MA0652.1/Jaspar(0.729) More Information \| Similar Motifs Found | motif file (matrix) |
| 57 | A C T G A G T C A C G T A T C G C T A G A C G T C T G A A G T C C A G T A C T G A C T G A C T G A C T G | 1e-17 | -4.106e+01 | 0.83% | 0.07% | 251.9bp (104.1bp) | MZF1/MA0056.1/Jaspar(0.592) More Information \| Similar Motifs Found | motif file (matrix) |
| 58 | A C T G A G T C G C T A A T C G C T G A G A T C C G A T | 1e-16 | -3.899e+01 | 40.07% | 33.25% | 329.7bp (144.7bp) | Smad3(MAD)/NPC-Smad3-ChIP-Seq(GSE36673)/Homer(0.764) More Information \| Similar Motifs Found | motif file (matrix) |
| 59 | A C G T A C G T C G T A A C G T A G T C A C G T A G T C A C G T A G T C A G C T A C G T A G T C A C G T | 1e-16 | -3.867e+01 | 0.47% | 0.01% | 117.2bp (109.8bp) | Gata4/MA0482.1/Jaspar(0.683) More Information \| Similar Motifs Found | motif file (matrix) |
| 60 | C A T G A T G C G C T A C T A G T A C G A T G C G A C T A C G T G A C T G A C T | 1e-16 | -3.817e+01 | 12.11% | 8.00% | 301.2bp (142.9bp) | Nr2e3/MA0164.1/Jaspar(0.620) More Information \| Similar Motifs Found | motif file (matrix) |
| 61 | A G C T A T G C C G A T A T C G T C G A T C A G A T C G T G A C A C T G A T G C T A G C | 1e-16 | -3.763e+01 | 7.66% | 4.46% | 307.6bp (138.5bp) | E2F1/MA0024.3/Jaspar(0.634) More Information \| Similar Motifs Found | motif file (matrix) |
| 62 | A C G T A G T C A C T G C T G A A C T G A C T G A C G T A C G T | 1e-14 | -3.281e+01 | 4.91% | 2.58% | 219.8bp (138.4bp) | RUNX2/MA0511.2/Jaspar(0.768) More Information \| Similar Motifs Found | motif file (matrix) |
| 63 | A C T G C G T A A C T G G A T C A G T C C G A T A C T G C T A G A G C T C G T A C T A G C G T A A G T C A G C T | 1e-13 | -3.120e+01 | 0.49% | 0.03% | 136.1bp (126.7bp) | ZEB1(Zf)/PDAC-ZEB1-ChIP-Seq(GSE64557)/Homer(0.504) More Information \| Similar Motifs Found | motif file (matrix) |
| 64 | C G A T A C G T G A C T A C G T C T A G A C G T C A T G A T C G A C G T A C T G A C T G A G C T A C G T A C T G | 1e-13 | -3.120e+01 | 0.75% | 0.09% | 164.6bp (175.4bp) | RUNX2(Runt)/PCa-RUNX2-ChIP-Seq(GSE33889)/Homer(0.642) More Information \| Similar Motifs Found | motif file (matrix) |
| 65 | C T A G G T A C G C T A T G C A T A C G A C G T G A T C A G C T T G C A T G A C A G T C C G T A T C A G | 1e-13 | -3.030e+01 | 0.78% | 0.11% | 192.4bp (141.9bp) | SD0002.1\_at\_AC\_acceptor/Jaspar(0.593) More Information \| Similar Motifs Found | motif file (matrix) |
| 66 | A T C G C G T A C G T A A T C G C T A G A T G C A C G T A C T G C G A T A G T C T A C G A C G T A G C T | 1e-12 | -2.799e+01 | 0.49% | 0.04% | 249.1bp (117.8bp) | RUNX-AML(Runt)/CD4+-PolII-ChIP-Seq(Barski\_et\_al.)/Homer(0.634) More Information \| Similar Motifs Found | motif file (matrix) |
